# Cost-effectiveness of bevacizumab plus paclitaxel versus paclitaxel for the first-line treatment of HER2-negative metastatic breast cancer in specialist oncology centers in France

**DOI:** 10.1186/s12885-019-5335-8

**Published:** 2019-02-11

**Authors:** Audrey Petitjean, Jayne Smith-Palmer, William Valentine, Bertrand Tehard, Stephané Roze

**Affiliations:** 1 HEVA HEOR, Lyon, France; 2 Ossian Health Economics and Communications GmbH, Basel, Switzerland; 3 Roche SAS, Boulogne Billancourt, France

**Keywords:** Cost, Cost-effectiveness, Breast cancer, Bevacizumab, France

## Abstract

**Background:**

Evidence from clinical trials suggests that the addition of bevacizumab to chemotherapy in the first-line treatment of patients with HER2-negative metastatic breast cancer improves progression-free survival (PFS) but not overall survival (OS). However, a retrospective analysis of real-world data from the French Comprehensive Cancer Centers (FCCC) through the Epidemiological Strategy and Medical Economics (ESME) Research Program, suggested that in this setting, the addition of bevacizumab may confer a significant benefit in terms of both PFS and OS. A cost-effectiveness analysis was performed to determine the cost-effectiveness of bevacizumab plus paclitaxel versus paclitaxel alone in the first-line treatment of HER2-negative metastatic breast cancer at specialist oncology centers in France.

**Methods:**

The analysis was performed using a three-state Markov model and clinical input data from *N* = 3426 HER2-negative metastatic breast cancer patients treated with bevacizumab plus paclitaxel or paclitaxel alone. The analysis was performed from a third party payer perspective over a 10-year time horizon; future costs and clinical outcomes were discounted at 4% per annum.

**Results:**

In the overall population, the addition of bevacizumab to paclitaxel led to incremental gain of 0.72 life years and 0.48 quality-adjusted life years (QALYs) relative to paclitaxel alone. The incremental lifetime cost of the addition of bevacizumab was EUR 27,390, resulting in an incremental cost-effectiveness ratio (ICER) of EUR 56,721 per QALY gained for bevacizumab plus paclitaxel versus paclitaxel alone. In a subgroup of triple negative patients the ICER was EUR 66,874 per QALY gained.

**Conclusions:**

The analysis indicated that the combination of bevacizumab plus paclitaxel is likely to be cost-effective compared with paclitaxel alone for the first-line treatment of HER2-negative metastatic breast cancer in specialized oncology centers in France.

## Background

In Europe, breast cancer is the most commonly occurring type of cancer in women [1], and accounts for approximately 12% of the total economic burden of cancer within the European Union [2]. Indeed, in 2009 the total annual cost of breast cancer in the EU was estimated at EUR 15 billion, including EUR 3 billion in drug costs [2]. In addition to the considerable economic burden, breast cancer is the fourth leading cause of cancer-related disability-adjusted life years [3]. In France, in 2015, there were an estimated 54,000 cases and 12,000 deaths from breast cancer, making it the leading cause of cancer-related mortality in women [4]. Moreover, a study from the Regional Institutional Registry from the Côte d’Or region showed that whilst almost half of breast cancer patients in this region were diagnosed with stage I breast cancer, 5.5% of women has already progressed to stage IV (metastatic) breast cancer at the time of diagnosis [5]. Further, in France an estimated 70% of women with metastatic breast cancer have HER-negative disease [6] and a regional study reports that 12% of metastatic breast cancers are triple negative [7].

One available treatment option for women with metastatic HER2-negative breast cancer is the vascular endothelial growth factor (VEGF) inhibitor bevacizumab in combination with paclitaxel. However, there has been controversy over the efficacy and cost-effectiveness of this regimen owing to a lack of a statistically significant benefit in overall survival (OS) compared with taxanes alone. This resulted in the initial Food and Drug Administration (FDA) approval of this regimen in 2008 being later rescinded in 2010 [8]. In contrast, in Europe bevacizumab initially received marketing authorization for use in combination with paclitaxel in metastatic breast cancer in March 2007, based on data from the E2100 trial [9]. The European Medicines Association (EMA) completed a review of bevacizumab in 2010, confirming that in metastatic breast cancer, the benefits outweighed the risks and stating the combination of bevacizumab plus paclitaxel “*remains a valuable treatment option for patients suffering from metastatic breast cancer*” [10].

However, clinical trials have consistently shown that the addition of bevacizumab to taxanes, in particular paclitaxel, confers a significant benefit in terms of progression-free survival (PFS) compared with taxanes alone [9, 11–13]. The combination of bevacizumab plus paclitaxel is approved for use in the EU as a first-line treatment for women with HER2-negative metastatic breast cancer [14]. Further, it is also included as a recommended regimen in both the European Society for Medical Oncology (ESMO) and National Comprehensive Cancer Network (NCCN) guidelines [15, 16]. Observational studies conducted in routine clinical practice have also been consistent with results from clinical trials in terms of PFS [17, 18]. Notably, however, in a recently published large scale observational study from France, Delaloge et al. reported a statistically significant improvement with bevacizumab plus paclitaxel versus paclitaxel alone for both PFS and OS [19].

The high cost of bevacizumab has further contributed to the controversy surrounding its use in metastatic breast cancer. In France, healthcare expenditure on cancer drugs accounts for approximately 3% of total healthcare spending [20]. One of the key principles of the French healthcare system as well as the French 2014–2019 Cancer Plan [21] is to provide universal access to innovation and quality medicines. The ESMO guidelines further supports this position for breast cancer patients specifically, stating that “*every advanced breast cancer patient must have access to optimal cancer treatment and supportive care according to the highest standards of patient-centered care*.” [15] However, continuing advances in treatment and the approval of new high cost regimens further increase the already high economic burden of this policy. As such, it is becoming increasingly important for oncology treatments to demonstrate cost-effectiveness, and in France, this is a mandatory consideration in terms of treatment and policy decision making for both payers and physicians. Cost-effectiveness analyses upon which payers decisions are frequently based typically utilize data from clinical trials, and these patient populations may not always be truly representative of the patient population encountered within routine clinical practice. Moreover, ESMO guidance also encourages real world studies to ascertain data on the performance of regimens within routine practice and the French Commission Évaluation Économique et de Santé Publique (Economic and Public Health Committee) also requires the current practice to be considered. The objective of the current analysis was to utilize recently published data from specialist oncology centers in the French Comprehensive Cancer Centers (FCCC) network to evaluate the cost-effectiveness of bevacizumab plus paclitaxel compared with paclitaxel alone for the first-line treatment of metastatic HER2-negative breast cancer in France. In France there are a total of 20 FCCCs, which together constitute the Unicancer network of centers and are non-profit centers exclusively dedicated to the treatment of cancer. The French Epidemiological Strategy and Medical Economics (ESME) database is an academic database of individual patient-level data on patients treated within FCCCs and approximately one-third of breast cancer patients in France are treated at FCCCs. Previously published real world observational data from the FCCC network were used to inform a cost-effectiveness model to provide estimates of the long-term clinical and economic outcomes associated with treatment with bevacizumab plus paclitaxel versus paclitaxel alone for the first-line treatment of HER2-negative metastatic breast cancer.

## Methods

### Time horizon, discount rate and perspective

The analysis was performed from a third-party payer perspective over a time horizon of 10 years, which was deemed sufficient to capture survival outcomes for the majority of patients with metastatic breast cancer. Future costs and clinical outcomes were discounted at a rate of 4% per annum in line with national recommendations [22].

### Model structure

**Fig. 1:**
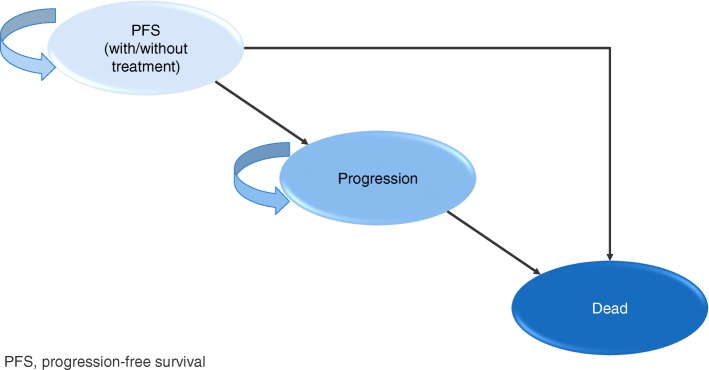
Schematic diagram of Markov model utilized in the cost-effectiveness analysis

The analysis was performed using a three-state model built in Microsoft Excel^®^. Details of the model have been previously published by Dedes et al. (2009) [23], but structurally the model is a 3-state Markov model with three mutually exclusive states (PFS, progression, which incorporates second-line and subsequent lines of treatment, and death) and at model entry all patients had stable/responsive disease (Fig. 1). The model was adapted to utilize a weekly cycle length (with half cycle correction). In the present analysis the approach used to estimate PFS and OS was that of partitioned survival analysis (area under the curve modeling) wherein PFS and OS curves are derived independently from analyses of each time to event endpoint [24].

The analysis compared the cost-effectiveness of bevacizumab plus paclitaxel versus paclitaxel alone as this was deemed to be the most relevant comparator with available data. Hormonal therapy was not considered to be a relevant comparator as hormonal therapy owing to usage at a different stage in the treatment pathway. Hormonal therapy is typically used first in hormone positive metastatic breast cancer in order to delay the time to onset of chemotherapy.

### Clinical input data

Survival functions at each time point were sourced from a high quality retrospective analysis of real world data from patients with HER2-negative metastatic breast cancer treated in specialist oncology centers using the ESME metastatic breast cancer (MBC) database (Table 1) [19]. The ESME database collects data from the patient electronic file, which contains the patient’s medical records, the pharmacy database containing treatment-related information as well as data used to inform a national level database relating to hospitalizations. Individual patient data utilized in the study by Delaloge et al. [19] were identified via International Classification of Disease codes for inpatient stays relating to metastatic breast cancer and/or pharmacy records or other databases specific to metastatic breast cancer. Additionally, Delaloge et al. report that in multivariate analysis (using a Cox model adjusted and stratified for prognostic factors of survival and potential confounders) the combination of bevacizumab plus paclitaxel was associated with median OS of 27.7 months, compared with 19.8 months for paclitaxel alone (in the overall population). Similarly, adjusted median PFS was 8.1 months for bevacizumab plus paclitaxel versus 6.4 months for paclitaxel alone [19]. A propensity-score matched analysis was performed in order to compensate for potential differences that may have occurred due to differences in prognostic factors between both groups. The base case analysis was performed in the overall population of HER2-negative patients. Additional subgroup analyses were performed in patients who were hormone receptor positive (HR+) and also in patients with triple negative breast cancer (TNBC) (i.e. HER2-negative, progesterone receptor negative and estrogen receptor negative).

**Table 1:** Baseline cohort characteristics

	Total (*N* = 3426)	Bevacizumab plus paclitaxel (*n* = 2127)	Paclitaxel (*n* = 1299)
Mean (SD) age, years	57.4 (12.4)	54.2 (11.2)	62.7 (12.5)
Female, n (%)	3391 (99.0)	2110 (99.2)	1281 (98.6)
Mean (SD) number of metastatic sites	2.1 (1.2)	2.0 (1.1)	2.2 (1.3)
De novo MBC, n (%)	663 (19.4)	347 (16.3)	316 (24.3)
Mean (SD) time (months) between initial diagnosis and metastatic diagnosis	63.6 (71.3)	61.2 (65.7)	67.6 (79.4)
Median time (months) between initial diagnosis and metastatic diagnosis	38.9	38.6	40.9
ER status			
Negative, n (%)	1042 (30.5%)	665 (31.4%)	377 (29.1%)
Positive, n (%)	2371 (69.5%)	1454 (68.6%)	917 (70.9%)
Not determined, n	13	8	5

*HR+* hormone receptor positive, *QALY* quality-adjusted life year, *TNBC* triple negative breast cancer

From Delaloge et al. 2016 [19]

**Fig. 2:**
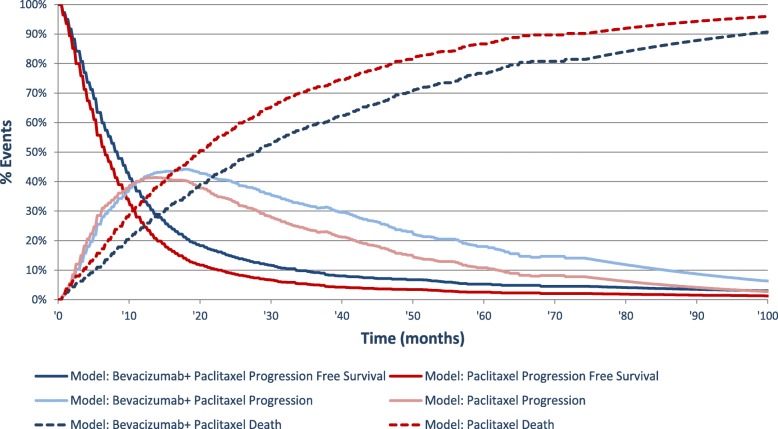
Markov trace and time in health states

The proportion of patients in each state was estimated at each model cycle based on survival curves for PFS and OS, and extrapolated beyond the follow-up time of the ESME MBC retrospective analysis on bevacizumab. Survival data for parametric extrapolation, using the least squares method, were adjusted for duration of pre-chemotherapy period, treatment period, grade, age at diagnosis of metastatic disease, type of metastases, number of metastatic sites, prior treatment history and contribution of site to the ESME MBC database (Fig. 2).

The adjustment of survival data based on prognostic factors prevented the calculation of survival based on the maximum likelihood method. The chosen option consisted of linearizing the survival function and then fitting using the least squares method. Not all survival distributions could be linearized, as such, Weibull and log logistic distributions were investigated and the OS, PFS and duration of treatment were fitted using this method. The distribution with the highest determination coefficient was selected for implementation in the model.

Data relating to the proportion of patients in each arm experiencing adverse events were sourced from the E2100 randomized controlled trial [9], and data on the proportion of patients receiving different second-line treatments were sourced from the Genactis study, a tracker study conducted routinely by Roche (Roche, data on file).

The methodology used by the ESME Research Program has been acknowledged as relevant and robust by the French Health Authorities [25].

### Costs and utilities

Costs accounted in the analysis included direct medical and non-medical costs including drug acquisition and administration costs for first-line treatment (based on treatment dosages sourced from the summary of product characteristics as these data were not available in the ESME study), adverse event costs, medical transport costs, follow-up and monitoring costs and costs associated with progression and treatment. Pharmacy costs were sourced from the BdM_IT CNAMTS database [26], costs for administration, travel, supportive care and adverse events were sourced from published literature [27–31]. Indirect costs associated with lost productivity were not included in the analysis. All costs are presented in 2016 EUR.

Health state utilities were a function of age, response to treatment, progression and chemotherapy-based adverse events and were sourced from a UK-based study [32]. The utility value applied in the PFS state was different in the two treatment arms owing to different frequencies of adverse events between arms. A disutility for hypertension (− 0.03) was also applied to the bevacizumab plus paclitaxel arm as this adverse event was not included in the Lloyd et al. [32] analysis, based on the findings of Nafees et al. [33] and the assumption that 14.8% patients in bevacizumab plus paclitaxel arm experienced hypertension (based on findings from the E2100 study [9]). In the base case no disutilities (as well as no costs) were applied for the adverse events of proteinuria, infections, allergic reactions, neuropathy, headache, or arthralgia were applied, owing to the absence of published data to quantify their impact on quality of life.

### Sensitivity analyses

A series of one-way sensitivity analyses were performed to determine key drivers of outcomes. Sensitivity analyses were performed around discount rates (1.5 and 6% per annum for both future costs and outcomes compared with 4% per annum in the base case), time horizon (5 years and 15 years compared with 10 years in the base case), as well as treatment and management costs (+/− 20%). Sensitivity analysis was also performed around the survival estimates using the upper and lower bounds of the 95% confidence intervals (CIs) around the point estimates for survival. Additional scenarios investigated included a scenario in which 100% patients in both treatment arms remained progression-free (i.e. 100% patients considered to be responders for the calculation of the utility value) and a scenario that permitted the sharing of bevacizumab vials, rather than single use, in the bevacizumab-treated arm. Probabilistic sensitivity analysis (PSA) was also performed (1000 iterations were run). In terms of distributions used for the PSA, for utilities/disutilities gamma distributions were used and for the progression-free state, progression state costs, administration costs and number of adverse events log normal distributions were used. It was not possible to include survival data in the PSA as the use of the least square method for extrapolation prevents the calculation of a covariance matrix for the coefficient of the survival distributions, thereby meaning that the PSA did not evaluate uncertainty around survival. Consequently, scenario analyses based on the fit of the 95% confidence intervals for survival were performed instead.

## Results

In the base case analysis for the overall population of HER2 negative patients with metastatic breast cancer, treatment with bevacizumab plus paclitaxel was associated with an incremental gain of 0.48 quality-adjusted life years (QALYs) relative to the use of paclitaxel alone (2.01 QALYs for bevacizumab plus paclitaxel versus 1.52 QALYs for paclitaxel alone). However, total lifetime costs were also higher for the bevacizumab arm (EUR 54,315 for bevacizumab plus paclitaxel versus EUR 26,925 for paclitaxel) resulting in an incremental cost-effectiveness ratio (ICER) of EUR 56,721 per QALY gained for bevacizumab plus paclitaxel versus paclitaxel alone (Table 2). The higher costs in the bevacizumab plus paclitaxel group were primarily driven by bevacizumab pharmacy costs as well as higher costs associated with disease progression, likely driven by longer overall survival (Table 3).

**Table 2:** Summary of base case results and subgroup analyses

	Bevacizumab plus paclitaxel	Paclitaxel alone	Difference
Life years gained			
Overall population	2.993	2.272	0.721
HR+	3.504	2.495	1.009
Triple negative	1.971	1.510	0.461
QALYs gained			
Overall population	2.006	1.523	0.483
HR+	2.327	1.674	0.652
Triple negative	1.341	1.015	0.327
Total lifetime costs, EUR			
Overall population	54,315	26,925	27,390
HR+	61,805	29,350	32,454
Triple negative	40,246	18,391	21,856
ICER, EUR per QALY gained			
Overall population		56,721	
HR+		49,749	
Triple negative		66,874	
ICER, EUR per life year gaine			
Overall population		38,003	
HR+		32,171	
Triple negative		47,447	

*HR+* hormone receptor positive, *ICER* incremental cost-effectiveness ratio, *QALYs* quality adjusted life years

**Table 3:** Breakdown of incremental costs according to treatment strategy in patients with HER2-negative breast cancer

Cost component	All patients		HR+ patients		TNBC patients	
	Paclitaxel alone	Paclitaxel+ bevacizumab	Paclitaxel alone	Paclitaxel+ bevacizumab	Paclitaxel alone	Paclitaxel+ bevacizumab
Adverse events, EUR	10	82	11	85	8	74
Progression cost, EUR	22,588	29,319	24,700	35,752	14,871	17,980
Supportive care in PPS, EUR	786	1951	868	2147	529	1411
Administration cost, EUR	3542	4078	3772	4231	2982	3691
Drug cost, EUR	0	18,885	0	19,590	0	17,091

*HR+* hormone receptor positive, *PPS* post-progression survival, *TNBC* triple negative breast cancer

All costs presented in 2016 EUR

The incremental benefit in terms of both life years gained and QALYs gained was most pronounced in the HR+ subgroup. In this subgroup, treatment with bevacizumab plus paclitaxel was associated with an incremental gain in quality-adjusted life expectancy of 0.65 QALYs relative to paclitaxel alone. The incremental cost of bevacizumab in this patient group was EUR 32,454, which led to an ICER of EUR 49,749 for bevacizumab plus paclitaxel versus paclitaxel alone in HR+ patients (Table 2).

In both treatment arms TNBC appeared to be associated with poorer outcomes. In the bevacizumab plus paclitaxel arm, TNBC patients had a quality-adjusted life expectancy of 1.34 QALYs (compared with 2.01 QALYs in the overall population). Similarly, for TNBC patients treated with paclitaxel alone quality-adjusted life expectancy was 1.02 QALYs (compared with 1.51 QALYs in the overall population). The lower quality-adjusted life expectancy in both arms resulted in a smaller incremental benefit with bevacizumab, resulting in an ICER of EUR 66,874 per QALY gained for bevacizumab plus paclitaxel versus paclitaxel alone.

**Fig. 3:**
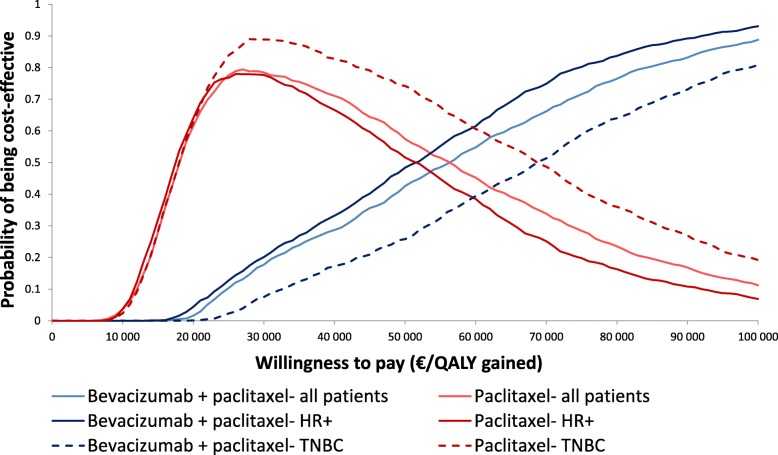
Cost-effectiveness acceptability curves for the treatment of metastatic HER2-negative breast cancer

A cost-effectiveness acceptability curve was plotted based on the PSA, which showed that the likelihood of bevacizumab plus paclitaxel being considered cost-effective in the overall population or patients with HR+ disease was between 40 and 50% at a willingness-to-pay threshold of EUR 50,000 per QALY gained (Fig. 3). However, when the willingness-to-pay threshold was increased to EUR 80,000 per QALY gained, the corresponding range increased to 70–80%.

Sensitivity analyses showed that the findings of the base case analysis were relatively robust (Table 4). Changes in discount rates and time horizon influenced the findings of the analysis as anticipated. Decreasing the time horizon of the analysis to 5 years increased the ICER to EUR 75,226 per QALY gained whilst extending it to 15 years resulted in the ICER decreasing to EUR 53,258 per QALY gained. Decreasing the discount rate for clinical outcomes to 1.5% per annum (compared with 4% per annum in the base case) led to an ICER of EUR 51,903 per QALY gained whilst increasing it to 6% per annum resulted in the ICER increasing to EUR 60,650 per QALY gained for bevacizumab plus paclitaxel versus paclitaxel alone. Additionally, the influence of assumptions relating to the OS was examined in scenarios in which the upper and lower 95% CIs of the OS benefit used. These had only a minimal effect on overall cost-effectiveness with the ICER decreasing to EUR 56,064 per QALY gained when the lower CI was used and increasing to EUR 57,173 when the upper CI for OS was used.

**Table 4:** Summary of scenario and sensitivity analysis (total cohort)

Scenario	Bevacizumab plus paclitaxel		Paclitaxel alone				ICER, EUR per QALY gained
	Life years	QALYs	Costs	Life years	QALYs	Costs	
Survival estimates							
Lower bound of 95% CI	2.996	2.008	54,163	2.269	1.521	26,874	56,064
Upper bound of 95% CI	3.193	2.151	57,041	2.457	1.654	28,634	57,173
Allowing sharing of vials	2.993	2.006	53,206	2.272	1.523	26,925	54,423
100% patients remaining progression-free^a^	2.993	1.970	54,315	2.272	1.550	26,925	65,196
Discount rate, future costs							
1.5% per annum	2.993	2.006	56,526	2.272	1.523	28,226	58,607
6% per annum	2.993	2.006	52,746	2.272	1.523	25,992	55,404
Discount rate, clinical outcomes							
1.5% per annum	3.162	2.113	54,315	2.372	1.586	26,925	51,226
6% per annum	2.874	1.931	54,315	2.201	1.479	26,925	60,650
Time horizon							
5 years	2.491	1.687	47,859	2.025	1.367	23,778	75,226
15 years	3.077	2.068	54,954	2.299	1.544	27,063	53,258

^a^100% patients in both arms remaining progression-free (i.e. for the calculation of the calculation of the utility value all patients are considered to be responders)

*CI* confidence interval, *ICER* incremental cost-effectiveness ratio, *QALY* quality-adjusted life year

## Discussion

In the current analysis, based on real world data, the combination of bevacizumab plus paclitaxel was associated with ICERs of EUR 38,003 per life year gained and EUR 56,721 per QALY gained compared with paclitaxel alone for the first-line treatment of patients with HER2-negative metastatic breast cancer in France. Subgroup analyses indicated that first-line treatment with bevacizumab plus paclitaxel was most cost-effective in patients with HER2-negative HR-positive metastatic breast cancer where the ICER was below EUR 50,000 per QALY gained and EUR 35,000 per life year gained for bevacizumab plus paclitaxel versus paclitaxel alone. Cost-effectiveness was also assessed in a subgroup of patients with TNBC, which represents approximately 15–20% patients with breast cancer [34]. Currently, the prognosis for patients with TNBC is poor, which is attributable to a combination of generally aggressive phenotypes and a lack of targeted therapies. In this subgroup, the combination of bevacizumab plus paclitaxel was associated with a gain in life expectancy and quality-adjusted life expectancy of 0.46 life years and 0.33 QALYs, respectively, as well as an ICER of EUR 66,874 per QALY gained, which although slightly higher than in the overall population may still bring an important benefit to TNBC patients at an acceptable cost.

For any drug used in oncology, the ability to identify patients more likely to respond well to treatment, and therefore take a more individualized approach to treatment may increase the cost-effectiveness of an intervention, although, for bevacizumab such data are currently lacking. In the AVADO trial, VEGF-A and VEGFR-2 were identified as potential biomarkers predictive of response to bevacizumab efficacy [35]. However, this finding has not been replicated in other studies [36]. Further research and the elucidation of any biomarkers predictive of response to bevacizumab-based treatment could be utilized to better individualize treatment options, which could in turn influence the cost-effectiveness of treatment with bevacizumab within the real world environment.

The current analysis is one of the first cost-effectiveness analyses of bevacizumab in HER2-negative metastatic breast cancer, and the first one in the French setting, to utilize clinical input data from a real-world environment. Important differences exist between the environments in clinical practice and clinical trials that can influence the mean effectiveness and in turn cost-effectiveness of interventions. In particular in clinical trials exclusion criteria frequently preclude the enrolment of patients with severe co-morbid conditions or poor performance status and patients aged > 65 are typically under-represented in many trials. Real world studies may subject to selection bias, but they do also provide an accurate representation of the patient population encountered in routine clinical practice. As a result, there is now an interest from payers for data relating to real-world effectiveness and cost-effectiveness as parameters such as effectiveness of treatment, adverse event rates, quality of life, adherence and medical resource utilization may differ between clinical practice and the clinical trial environment [37, 38].

Recently, van Kampen et al. used real-world data to examine the cost-effectiveness of bevacizumab in HER2-negative metastatic breast cancer in the Netherlands [39]. In their analysis, van Kampen et al. reported an ICER of EUR 155,261 per QALY gained, which is substantially higher than that reported in the present analysis for the French setting. Clinical input data were sourced from a total of 62 patients (*n* = 33 treated with bevacizumab plus a taxane and *n* = 29 treated with a taxane alone) treated in the Netherlands, with the relative effectiveness estimates for PFS and OS sourced from the E2100 trial [9]. In contrast, real world data used in the current analysis were sourced from a much larger group of patients (*n* = 2127 treated with bevacizumab plus paclitaxel and *n* = 1299 treated with paclitaxel alone) and utilized real-world OS and PFS data [19]. The authors of the French study suggest that real-world data should be interpreted with caution owing to the potential for bias in treatment assignment based on patient and/or disease characteristics. However, the findings of the French analysis, in particular those relating to OS, remained significant in both multivariate-adjusted and propensity score matched analyses. It should be noted that the propensity score matching adjusts for observed confounders but not unobserved ones. It should also be noted that in the Netherlands the ex-factory price of bevacizumab is approximately 10% higher than in France, which should also be taken into account when comparing the findings of analyses from these two countries [40].

The analysis of Delagoge et al. is among the first to report a significant improvement in OS for patients who initiated first-line treatment with bevacizumab [19]. In clinical trials, the OS benefit conferred by bevacizumab consistently failed to demonstrate statistical significance. Although, in a pooled analysis of the E2100, AVADO and RIBBON-1 trials, Miles et al. noted that 1-year OS was consistently higher in patients treated with bevacizumab compared with chemotherapy alone and suggested that patients at high risk of rapid progression or poor prognosis may derive the greatest benefit from bevacizumab-containing treatment [41]. However, it should be noted that whilst OS is often regarded as the “gold standard” in terms of endpoints, in both real world studies and clinical trials of first-line treatments, OS may be confounded by the influence of subsequent lines of treatment at progression and should therefore be interpreted with caution.

Whilst there is no formal willingness-to-pay threshold in France, in oncology a willingness-to-pay threshold of USD 100,000 per QALY gained is a commonly cited benchmark in US-based analyses [42, 43]. Similarly, in the Netherlands a threshold of up to EUR 80,000 is applied for severe conditions [44] and a recent analysis of the UK-based cancer drugs fund (CDF) suggested that the willingness-to-pay threshold of the CDF was in excess of GBP 200,000 per QALY gained for novel cancer treatments, which is substantially higher than commonly cited thresholds for the National Institute of Health and Care Excellence [45]. In France, however, no formal willingness-to-pay threshold exists with the economic implications of new interventions considered on an individual basis. In the present analysis, the ICER in all subgroups was under EUR 70,000 per QALY gained, and therefore substantially under commonly cited willingness-to-pay thresholds for oncology treatments across a number of countries including the US, UK and the Netherlands. The ICER reported here is also relatively low for an oncology treatment suggesting that the combination of bevacizumab plus paclitaxel represents a cost-effective treatment option for women with metastatic breast cancer. For example, in a US-based analysis of cost-effectiveness studies, the mean ICER for oncology drugs was USD 138,582 per QALY gained, compared with USD 49,913 for non-cancer drugs. Moreover, only 45% of ICERs for oncology drugs were below USD 50,000 per QALY gained, compared with 72% for non-cancer drugs [46]. In addition, the relative benefits in terms of the incremental gain is quality-adjusted life expectancy associated with the addition of bevacizumab to treatment for HER2-negative breast cancer is broadly in line with those reported for other novel oncology treatments. In particular, in a 2011 review by the Scottish Medicines Consortium (SMC) reported that the mean incremental QALY benefit provided by new treatments for use in advanced/metastatic cancer was 0.51 QALYs; however, it should be noted that this figure is likely to be based largely on data from clinical trials rather than routine clinical practice [47]. The incremental QALY gain in the overall population in the present analysis was 0.48 QALYs. In their Netherlands-based real-world analysis van Kampen et al. reported a QALY gain of 0.36 QALYs, with a lower incremental gain of 0.19 QALYs reported for their trial-based analysis, based on data from the E2100 trial [39]. Notably, Refaat et al. report a QALY gain of 0.37 QALYs for bevacizumab plus paclitaxel versus paclitaxel alone in another analysis, also utilizing clinical input data from the E2100 trial [48]. Taken together, these data suggest that the addition of bevacizumab to first-line chemotherapy in HER2-negative metastatic breast cancer patients may be more cost-effective in the real-world environment than in clinical trials. The reason for this is unclear, although one potential explanation is that it may be due to more judicious selection of patients for bevacizumab treatment in the real-world environment.

The current analysis is associated with a number of limitations. In particular, clinical input data for OS relied on the extrapolation of data from adjusted Kaplan-Meier curves: however, in the absence of available long-term data the approach used represents one of the best available proxies. Additionally, clinical input data were sourced from a retrospective observational study and there are limitations that are inherent in this type of study design. These include the potential for selection bias as in a real-world setting baseline patient and disease characteristics such as performance status may be important drivers of treatment decisions [19]. In addition, the current analysis only compared two available treatment options for patients with HER2 negative metastatic breast cancer. In Europe, bevacizumab is also indicated in combination with capecitabine for the first-line treatment of HER2 negative metastatic breast cancer in women who cannot be treated with taxanes or anthracyclines. Similarly, other taxanes or anthracylclines are also used as monotherapy as first-line treatment of metastatic breast cancer. Cost-effectiveness analyses of bevacizumab plus paclitaxel (or capecitabine) represents versus other comparators represents a potential avenue for future cost-effectiveness analyses.

## Conclusions

Recent real-world data from specialist oncology centers in France suggest that in this setting, the combination of bevacizumab plus paclitaxel confers a significant advantage in terms of both PFS and OS relative to paclitaxel alone. The findings of the cost-effectiveness analysis performed using this real-world data suggests that in France, the addition of bevacizumab to paclitaxel relative to paclitaxel alone is likely to represent a cost-effective treatment for patients with HER2-negative metastatic breast cancer, particularly in patients with TNBC.

## Abbreviations

CDF: Cancer drugs fund
CI: Confidence interval
EMA: European Medicines Association
ESME: Epidemiological Strategy and Medical Economics
ESMO: European Society for Medical Oncology
FCCN: French Comprehensive Cancer Centers
FDA: Food and Drug Administration
HER2: Human epidermal growth factor receptor 2
HR+: Hormone receptor positive
ICER: Incremental cost-effectiveness ratio
MBC: Metastatic breast cancer
NCCN: National Comprehensive Cancer Network
OS: Overall survival
PFS: Progression-free survival
PSA: Probabilistic sensitivity analysis
QALY: Quality-adjusted life year
SMC: Scottish Medicines Consortium
TNBC: Triple negative breast cancer
VEGF: Vascular endothelial growth factor
